# Soy Preparations Are Potentially Dangerous Factors in the Course of a Food Allergy

**DOI:** 10.3390/foods8120655

**Published:** 2019-12-07

**Authors:** Anna Jędrusek-Golińska, Dorota Piasecka-Kwiatkowska, Paulina Zielińska, Magdalena Zielińska-Dawidziak, Krystyna Szymandera-Buszka, Marzanna Hęś

**Affiliations:** 1 Department of Gastronomy Science and Functional Food, Poznań University of Life Sciences, Wojska Polskiego 28 str., 60-637 Poznań, Poland; anna.jedrusek-golinska@up.poznan.pl (A.J.-G.); marzanna.hes@up.poznan.pl (M.H.); 2 Department of Food Biochemistry and Analysis, Poznań University of Life Sciences, Mazowiecka 48 str., 60-623 Poznań, Poland; pau.gorecka@gmail.com (P.Z.); magdalena.zielinska-dawidziak@up.poznan.pl (M.Z.-D.)

**Keywords:** immunoreactivity, soy preparations, consumer awareness, qualitative research

## Abstract

The special properties of soy preparations make them common additives for food production and can be dangerous for sensitive individuals. Our aim was to check consumers’ awareness of potential risks of soy preparations added to numerous food products, depending on respondents’ education, and to evaluate immunoreactive properties of chosen soy preparations. A personal questionnaire was used. Respondents (*n* = 251) were aged 23–28 years old, lived in Poland, and were graduates or students in their last year of food technology, medicine, and university of technology. The slot blot and Western blotting methods were used to mark immunoreactivity of soy preparations. It was shown that most respondents often or usually read labels of food products they buy. The surveyed indicated protein is the allergenic component in soy. Almost half of them were of the opinion that hydrolysis removes the allergenic properties of soy. Most of the medical students surveyed thought that people allergic to soy may consume products that contain soy preparations. The analytical results indicated that soy preparation contained protein fractions that were immunoreactive with sera of allergenic patients. It was proven that preparations, even hydrolysates, contain immunoreactive proteins that may be the source of hidden allergens, even though they are not recognized as dangerous by well-educated respondents.

## 1. Introduction

Soy seed is a raw material for obtaining soy flour as well as various preparations including textured soy protein (TSP), soy protein concentrates (SPC), soy protein isolates (SPI), protein hydrolysates, and fermented products in which proteins and their structures may undergo various modifications [1,2]. Due to its special properties, related to hydration (solubility, water, and fat absorption), rheological characteristics (elasticity, viscosity, adhesiveness, jellying, and aggregation), and to protein surface features (foaming and emulsifying activities, whippability, and formation of protein-lipid films), these preparations are successfully used in food production. They are used in many food systems: meat and dairy, bakery and pasta, infant formulas, low viscosity (beverages), and high viscosity products (retortable sauces, mayonnaise, desserts) and confectionery [1,3].

Soy additives used in meat processing contribute to better binding of water and fat in the product. The emulsifying properties of soy proteins stabilize the ingredients of meat stuffing into a homogeneous system. Soy proteins also improve the consistency and structure of finished products as a result of the formation of strong gels [1,4]. In addition, they reduce production costs while maintaining a high quality and increasing the efficiency of products, especially medium-ground and finely-ground sausages [5]. The addition of soy protein also allows a consumer to receive new, dietary meat products with an increased content of total protein. Soy protein concentrates and isolates, due to their low viscosity, are used for the production of infant formulas, milk replacers, and creamers. They are also incorporated into emulsion-type cheeses, bread, and even doughnuts [3,6].

Due to the advantages of the use of soy additives in food production, they are used in an almost unlimited range. On the other hand, soy is one of the eight most dangerous food allergens. Hence, soy preparations used in food production can be a source of a “hidden allergen.” Anibarro et al. [7] analyzed 530 cases of allergic reaction and noted that 22.5% (119 persons) were caused by “hidden allergens.” They also observed that, in the case of people allergic to soy, the symptoms caused by the presence of additives were even more frequent—in as many as 39% of respondents, most often after consuming boiled ham, sausages, cheese puffs, precooked dishes, desserts, and gravy [7].

Allergic reactions after consuming food containing soy may also appear in people allergic to other legumes [8,9,10,11]. They were also observed in people with atopic diseases and those allergic to cow’s milk [12]. An increase in allergic symptoms occurring after eating soy is also noted in people who are allergic to birch, especially during the pollination period [13,14,15].

Allergenicity of food compounds, including preparations obtained from soy, may be decreased, increased, or unaltered, depending on the molecular characteristics of the allergen food protein, the type of processing, and the intensity of treatment [16]. Generally, heat, pressure, ultrasounds, and radiation (physical processes) can mask, expose, or destroy conformational epitopes of food proteins in the way of altering their secondary and tertiary structures. Fermentation and enzymatic hydrolysis (biochemical processes) may affect linear epitopes [2,16,17]. It can be assumed that soy protein hydrolysates usually added to food in a low amount (up to 3%) do not cause a significant increase in product immunoreactivity, but this has to be confirmed only by laboratory tests. Considering that, so far, no threshold doses that cause an allergic reaction have been established, it is not simple or obvious [18].

On the other hand, due to both the increase in the number of diagnosed allergy cases and consumers’ awareness of food allergy risk, requirements for labeling substances that cause allergies and intolerance reactions have been introduced. Mandatory labeling of food allergens allows persons suffering from these to make informed choices. Nevertheless, there are some gaps in the current legislation, including a lack of allergen threshold levels for coincidental contamination, which results in preventive labeling [19,20]. However, an additional form of voluntary labeling (termed precautionary allergen labeling), which should prevent exposure to traces of an allergen present in food due to cross-contamination during production, has led to a situation where such warnings are used almost everywhere. This, in turn, causes confusion among consumers regarding their significance [21]. Zurzolo et al. [22] showed that the wording “may contain traces” was the most commonly heeded statement (86% of allergic consumers avoided foods with this statement), followed by “manufactured on shared equipment” (79%), and “manufactured in a facility that also processes …” (64%). Such statements are not clear and they do not always assess the actual risk. Therefore, it is vital and necessary to study the immunoreactive properties of soy preparations in order to be able to responsibly assess their safety for allergic consumers. Only based on such analytical results is it possible to reliably and unequivocally label products containing such additives.

## 2. Aim of the Study

The aim of the study was to check consumers’ awareness of potential risks of soy preparations frequently added to numerous food products, depending on respondents’ education, and to evaluate immunoreactive properties of chosen soy preparations on the Polish market.

## 3. Materials and Methods

### 3.1. Questionnaire Survey

#### 3.1.1. Individuals Participating in the Survey

All respondents (*n* = 251) living in Poland (Wielkopolska region) were surveyed using a personal questionnaire in November 2017. Their selection was intentional. The inclusion criteria were: a young age (aged 23–28 years), being in their last year of studies or higher education in the field of engineering management, food technology, or medicine, no allergies to foods, and giving answers to all questions in the questionnaire. Women accounted for 32.3% of the respondents (Table 1). The average age of respondents was 25. To investigate the impact of the type of education on some attitudes toward soy protein preparations and awareness about their possible immunoreactivity, respondents were divided into three groups: engineers (E-people with bachelor‘s or master‘s engineering in management degrees, *n* = 101), food technologists (F-people with bachelor of engineering or master of engineering degree in food technology, *n* = 100) and medics (M-students in their last year and medical graduates, *n* = 50).

#### 3.1.2. Interview Questionnaire

The questionnaire was created in the Department of Gastronomy Science and Functional Foods, Poznan University of Life Sciences. It was pre-tested individually by 19 respondents in the presence of the investigator. The survey took the form of an interview questionnaire with respondents in their place of work/study. The questionnaire was anonymous and voluntary, did not interfere with the respondent’s other duties, and ensured freedom of expression and comfort. The interviewer explained any potential doubts.

Even if the questionnaire included some additional questions, mainly regarding the frequency of consumption and the health and technological benefits/disadvantages of soy and preparations, only those significant for the presented work are discussed in this paper.

### 3.2. Analytical Study

#### 3.2.1. Materials

(A) The soybean seeds (*Glycine max*, Augusta variety) were obtained from the Department of Genetic Plant Breeding, Poznan University of Life Sciences. The seeds were ground and not degreased.

(B) Soy functional additives used in the meat industry.

1. Soy protein isolates:
  (1) IP 9000A, Gushen, China
  (2) Profam 648, ADM USA
  (3) IBS (Soy Protein Isolate), Guscen, China
2. Soy protein concentrate: Sol Pro 110 Solbar, Israel
3. Soy protein acid hydrolysates (SHB) from Polish company “PAULA Ingredients”:
  (1) SHB Paula,
  (2) SHB Paula Plus,
  (3) SHB KP,
  (4) SHB HVP.

Soy additives were in the form of powder with a meat aftertaste. 

The commercial hydrolysates were made with the addition of potato maltodextrin as a carrier. Moreover, SHB Paula Plus contains yeast extract, SHB KP, and SHB HVP peptides from maize and rapeseed.

#### 3.2.2. Patients

The sera of three patients that had allergic sensitization to soy from SNOZ Alergologia Plus Center for Diagnosis and Treatment of Allergy Therapy in Poznań (Poland) with a diagnosis of IgE-mediated soy allergy were used for investigation. Moreover, patients described additional pollen-related food allergies. The concentration of the allergen-specific IgE antibody (kUa/L) was estimated by the Polycheck test (Biocheck) and grade, according to the semiquantitative RAST scale as follows: 0 (below 0.33 kUa/L), 1 (0.33–0.7 kUa/L), 2 (0.7–3.4 kUa/L), 3 (3.5–17.4 kUa/L), 4 (17.5–52.4 kUa/L), 5 (52.5–100 kUa/L), and 6 (above 100 kUa/L).

Bioethical Commission at Poznan University of Medical Sciences (Poland) accepted the application for permission to carry out this experiment (No 1112/12). Allergenic characteristics of patients’ sera used in research according to the RAST scale was as follows.

1. Serum A: Soy − 2, Birch and oak − 3, Alder and hazel − 2, Grass-mix − 2, Yeast − 2, Potatoes − 2, Hazelnuts 1
2. Serum B: Soy − 2, D. pteron + D. farinae − 2, Bovine serum albumin − 1, α-Lactoalbumin − 1
3. Serum C: Soy − 2, Timothy − 6, Asp. fumigatus − 3.

#### 3.2.3. Protein Content

The protein contents in soy seeds, isolates, concentrates, and hydrolysates were determined by means of the Kjeldahl method [23]. Nitrogen content was calculated into protein content with the conversion factor 6.25. 

#### 3.2.4. Protein Extraction

Protein from analysed samples was extracted with 0.1 M TBS-HCl pH = 8.6 with additives: 2% of 2-mercaptoethanol, 2% SDS, 0.05% Tween 20. The samples were mixed at room temperature with the proportion w:v 1:10 and shaken for 1 h and then centrifuged (5000× *g* 4 °C). The extraction was made in triplicate and then pooled into one sample. 

#### 3.2.5. SDS-PAGE (Sodium Dodecyl Sulfate—Polyacrylamide Gel Electrophoresis)

The electrophoresis was running at 4 °C, at a constant voltage of 90 V in the stocking gel, and 170 V in the resolving gel. The gels were stained with Coomassie Brillant Blue R-250 and analysed using CLIQS (TotalLab Quant, Great Britain, Newcastle, UK).

#### 3.2.6. Western Blotting

Proteins separated with SDS-PAGE were transferred to the PVDF membrane (Immobilone IPVH00010 Merck Millipore Ltd., Darmstadt, Germany). Next, the membranes were blocked with 0.01 M TBS (Tris-Buffered Saline), pH 7.4 containing 1% BSA (Bovine Serum Albumin) (Sigma A7906, Saint Louis, MO, USA) for 1 h. The detecting antibody was the polyclonal anti-soy (Sigma S2519) diluted 1:1000 in blocking the TBS-BSA buffer. The incubation with the antibody lasted one hour. After fivefold washing, membranes were incubated for 1 h with anti-rabbit IgG (γ-chain specific) mouse monoclonal antibody conjugated to alkaline phosphatase (Sigma A2556) diluted 1:8000 with blocking buffer containing 0.05% Tween 20 (Sigma P9416). Membranes were washed five times and then the substrate (BCIP/NBT- Clbiochem, San Diego, CA) was applied for 20 min. The reaction was stopped and the membranes were air-dried in the dark and, afterward, analysed with the CLIQS program (TotalLab Quant, Great Britain, Newcastle, UK).

#### 3.2.7. Slot Blotting

The extract from IBS (100 μL) was immobilized on the PVDF membrane with a Slot-Blotter (Roth). The immunostaining procedure was similar to Western blotting, except that it used antibodies. The detecting one was diluted 1:20 sera of allergenic patients, whereas the second antibody was diluted to 1:1000 mouse monoclonal anti-human IgE labeled by alkaline phosphatase (Sigma A3076).

### 3.3. Statistical Analysis

A chi-squared test was used (Statistica 10.0) for the examination of the influence of the education type on the type of respondents’ awareness and attitudes. The question in which the Likert scale was used was presented using correspondence analysis. 

The protein content results were subjected to the analysis of variance (ANOVA). The significance of ANOVA was checked with the F-test. In case of significant differences, a post-hoc analysis (Tuckey’s test) was performed. The data were expressed as an average ± standard deviation.

## 4. Results

### 4.1. Questionnaire Survey

In our study, 92% of respondents declared to be reading labels of food products, with 45.8% of respondents always checking the content of the purchased food products, and 46.2% checking it ‘usually’ (Table 2). This result is similar to the finding obtained by Bandara et al. [24], who observed that 98% of the population surveyed read labels, and a different result was obtained by Goyal and Deshmukh [25], where 52.5% did not read labels. It is likely related to the choice of the group surveyed. We questioned persons with higher education only, including food technologists and medics, which might translate to a more frequent use of labels as a source of information about the food products. We observed significant differences between the groups studied (*p* < 0.001)–engineers read labels the least frequently (82.1% vs. 96% among medics and 100% among food technologists).

The respondents were also asked which of the base ingredient of soy they deemed the most allergenic. It was a way to introduce further issues in the questionnaire, regarding methods that might lower the allergenic properties of soy. The participants pointed at protein as the most often allergic component (68.5%) or declared to have no knowledge concerning that subject (23.5%). Based on a chi-square test, a significant influence on the type of education on answers given was shown. The highest percentage of correct indications was observed among food technologists (93%). It was surprising that medics indicated protein as the factor responsible for the allergenic properties of soy the least often (38%) and chose the answer ‘I do not know’ most often (40%). More than one-fifth (22%) of that group claimed it was carbohydrates included in soy that caused the allergic reaction. 

The education system for doctors in Poland provides general education in the course of studies and then assumes it will be intensively deepened during the training for a medical specialty. The medics we interviewed were not allergy specialists. Nevertheless, their knowledge about soy components responsible for its allergenic properties was not sufficient. As a consequence of this lack of knowledge, they may not be acquainted with methods of changing/lowering the immunoreactive properties of soy. This, in turn, leads to their lack of awareness of patients’ allergic reactions to soy. It is vital to pay attention while preparing meals and buying food products. This awareness was checked in the next question (Table 3). Among the listed processes, hydrolysis and thermal processing were most often indicated by respondents as processes that may lower the allergenic properties of food products (meaning—proteins present. However, not all interviewees were aware of the fact that it is proteins that are mainly responsible for the allergic reaction). This opinion was expressed by less than half of respondents (47% and 46%, respectively). The chi-square test showed a significant influence on the type of education regarding the type of answers given: hydrolysis as a process leading to a decrease of the food’s allergenic character was indicated by 62% of food technologists and 48% of medics, whereas thermal processing was indicated by 48% of food technologists and 70% of medics, respectively. More than 30% of engineers answered these questions positively. Respondents were also not certain whether genetic modification may result in soy of lower immunoreactive properties. Fewer than 23% of the surveyed, including over half of the engineers, could not answer the question ‘what processes may lower the allergenic character of soy.’ 

Another question included in the questionnaire concerned whether persons’ allergic reactions to soy might consume food products that include soy preparations. As stated in the introduction to this paper, due to good technological properties, soy and its preparations are added to a large range of food products.

**Figure 1:**
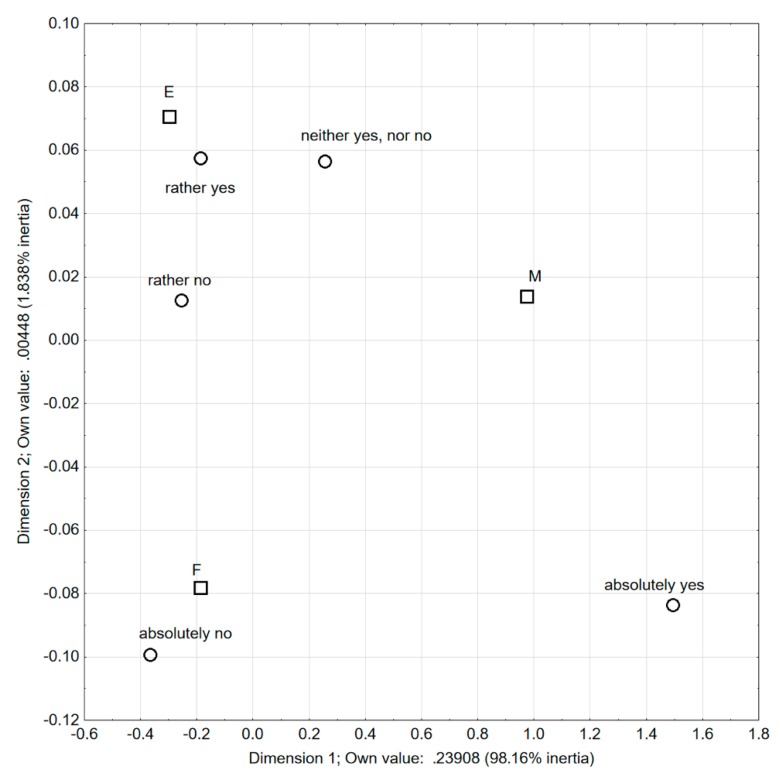
Respondents’ answers to the question: Do many persons allergic to soy consume products with added soy preparations? (correspondence analysis).

In the question, the 5-point Likert scale was used, with the possibility to answer ranging from ‘absolutely no’ to ‘absolutely yes’. For the analysis of the obtained results, the correspondence analysis was used (Figure 1). Based on it, certain general observations may be formed on the character of dimensions, based on the claim for which side of the axis points are given. Points representing the categories ‘absolutely yes’ and ‘neither yes nor n*o*’ (regarding the question ‘Can persons allergic to soy consume products with added soy preparations?’) are found on the right of the first axis, which is similar to the group *‘medics’* (M). Therefore, a conclusion may be drawn that the first axis differentiates between the category *‘absolutely yes’* and *‘neither yes nor no’* from other opinions concerning this matter. In addition, *‘medics’* were different from engineers (E) and food technologists (F) with respect that, among them, there were relatively more people completely convinced that people allergic to soy may consume food with soy preparations added, or who had no opinion on that issue.

### 4.2. Analytical Study

The interest in soy as a raw material for food production is directly related to its protein content (around 40%) with an extremely favorable amino acid composition. The protein content of isolates and concentrates is even higher at around 80–90% and 60–80%, respectively [3]. 

The protein content determined by the Kjeldahl method in the studied soy seeds, isolates, concentrate, and hydrolysates showed a typical value (soy seeds Augusta 34.18 ± 2.4%, IP9000A 81.41 ± 2.8%, Profam 648 84.48 ± 2.1%, IBS 85.30 ± 1.9%, Sol-Pro110 61.09 ± 1.5%, SHB Paula 7.06 ± 0.4%, SHB Paula Plus 13.97 ± 0.7%, SHB KP 17.02 ± 0.7%, and SHB HVP 6.25 ± 0.5%). The highest protein content was measured in the isolates (average around 84%) and statistically significant differences between samples were not noted. A slightly lower protein content was observed in the concentrate (61%). The lowest was noted in the hydrolysates, but, between these analysed samples, there were statistically significant differences. The protein contents in SHB KP and SHB Paula Plus were even over two times higher (2.7 and 2.2, respectively) compared to the SHB HVP. SHB KP is a concentrated hydrolysate. Therefore, it was not surprising that it contained more protein compared to other analysed samples. A higher protein content in SHB Paula Plus was seen likely due to the fact that, in addition to hydrolysed soybean, it contained another source of protein, i.e., yeast extract.

**Figure 2:**
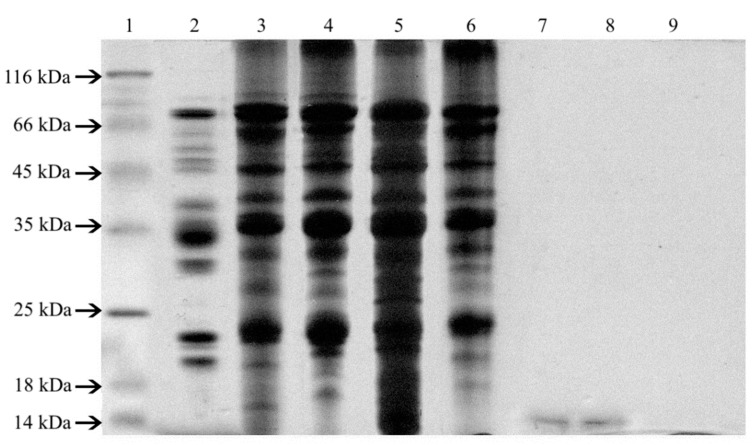
SDS-PAGE profiles of extracts obtained from soy seeds, isolates, concentrate, and hydrolysates: MW- molecular weight marker, 1- soy seed Augusta, 2–IP 9000A, 3–Profam 648, 4–IBS, 5–Sol-Pro 110 6-SHB Paula, 7–SHB Paula Plus, 8–SHB KP, and 9–SHB HVP.

The diversity of protein fractions content in soy seeds, isolates, and concentrates was studied by SDS-PAGE electrophoresis (Figure 2). Eleven protein fractions with molecular weights of 16–18, 20–22, 26–28, 34, 50, 55, 65, and 82 kDa were identified in extracts obtained from soybeans. An additional peptide with molecular weight over 100 kDa was noted in soy functional additive isolates and concentrates. Only one protein fraction with a molecular weight of approximately 14 kDa was identified in the patterns of HBS Paula and HBS Paula Plus hydrolysates and none in the other two analyzed hydrolysates. The molecular weights of separated proteins calculated on the basis of densitometric analysis of electrophoregram suggested the presence of allergenic peptides in all analysed samples.

**Figure 3:**
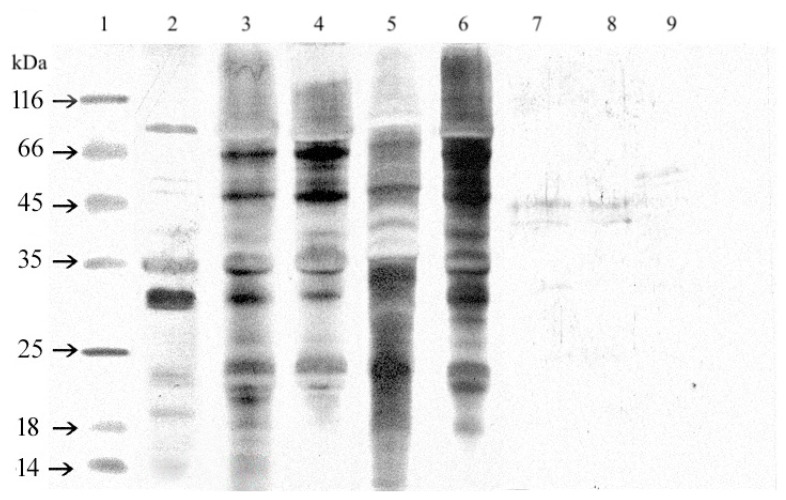
The immunoblot of extracts obtained from soy seeds, isolates, concentrate, and hydrolysates under reducing conditions with a polyclonal anti-soy antibody (Sigma S2519). MW- molecular weight marker, 1- soy seed Augusta, 2–IP 9000A, 3–Profam 648, 4–IBS, 5–Sol-Pro 110 6–SHB Paula, 7–SHB Paula Plus, 8–SHB KP, and 9–SHB HVP.

Immunoreactivity of proteins separated by SDS-PAGE was analyzed by immunoblotting and staining with Sigma (S2519) polyclonal anti-soy antibody. The obtained results are presented in Figure 3. Western blot analysis with polyclonal Sigma anti-soy antibodies confirmed that all functional additives, isolates, and concentrates contained proteins with antigenic properties relative to these antibodies. The molecular weight of recognized proteins was similar to the allergens: Gly m 3 or Gly m 8 (14 kDa), Gly m 4 (16–18 kDa), STI (20–22 kDa), Gly m Bd 28 K (26–28 kDa), Gly m Bd 30 K (31–34 kDa), Gly m 5–β- subunit of β-conglycinin (50 kDa), subunit of G5 Gly m 6 (55 kDa), Gly m 5–α- subunit of β-conglycinin/Gly m Bd 60K (65 kDa), and Gly m 5-α’-subunit of β-conglycinin (82 kDa) [8,26,27,28,29].

Proteins in hydrolysates were less immunoreactive. There were fractions recognized by the polyclonal anti-soy antibody in only three samples. The immuno-patterns of SHB Paula and SHB Paula Plus were similar. The molecular weight of recognized protein fractions was 40 and 45 kDa, whereas, in SHB KP, the anti-soy antibody identified only one protein band with molecular weight around 55 kDa. In SHB HVP, no immunoreactive peptide was recognized.

**Figure 4:**
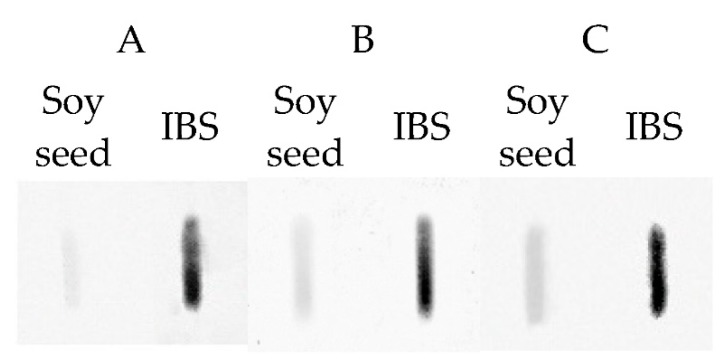
Human IgE-binding to proteins from soy seed and Soy Protein Isolate (IBS). A, B, C-three different sera of patients had sensitization to soy.

Immunoreactivity of extracts obtained from soy seed isolates with the highest protein content was additionally analyzed by Slotblot with sera of patients’ allergies, among others, to soy (Figure 4). A serum of a non-soy allergic patient in which IgE-binding to proteins was not demonstrated was used as a control. In the experiment, the capacity of human IgE-binding to proteins, which is related to the allergenic properties of the tested samples, was analyzed. The obtained pattern confirmed the existence of allergenic properties of the extract regardless of the serum. Immunoreactivity of isolates was even higher compared to soy seed since the protein content in isolates was higher compared to the seed. Therefore, even if soy isolates are added to products as additives in a small amount, they can still be dangerous to people with allergies. 

## 5. Discussion

Consumers’ expectations toward the nutritious aspect of food are growing. In this context, food product labels play a significant role in providing consumers with adequate nutrition-related information, which facilitates making healthy and safe choices when buying food products [30]. Food allergen labeling is also an important tool in reducing the risk of exposure and in preventing anaphylaxis for individuals with food allergies [31]. It is especially important in case of masked allergens. The necessity to educate patients as well as clinicians about relevant sources of allergens and cross-reactive allergens to prevent accidental reactions was emphasized by Baker et al. [32] and Jędrusek-Golińska et al. [33]. 

According to Bandara et al. [24], the major reasons to examine the food labels by consumers are: to evaluate the suitability of the food product for vegetarians, to avoid diseases related to food, religious reasons, and to check whether the food is grown organically or not. However, this information is often not fully used by consumers [20]. There are reports proving that information included in labels is complex (especially in view of the fact that products are not staple goods but highly-processed products of added value) and they do not always fulfill the potential of effective communication [34,35,36,37]. A large amount of information included in labels discourages food buyers from checking the information included there. A review carried out by Soederberg Miller and Cassady [34] showed that nutrition knowledge provides support for food label use. 

The possibility to lower immunoreactive properties with the use of various technological efforts was the subject of numerous papers [2,8,17,38]. Generally, it may be stated that processing does not fully remove the allergenic potential of allergens. Song et al. [39], using sera of soy allergic patients, indicated that soy protein isolate (SPI) and concentrate (SPC) showed less IgE-binding capacity than soy flour. The IgE-binding capacity of tofu was about 20-fold higher than that of soymilk [39]. 

Currently, only hydrolysis and fermentation are recognized as methods that potentially reduce allergic reactions to such an extent that symptoms would not be induced, while other methods are promising but require more studies [2]. Hydrolysis is the most common method used for obtaining hypoallergenic formulas. The enzymatic hydrolysis of glycinin and β-conglycinin with tryptic and peptic enzymes showed that, at a low pH, glycinin was denatured and more susceptible to hydrolysis, while β-conglycinin was denatured at higher temperature and became more hydrolyzed in contrast to glycinin, which was not affected. However, the IgG-binding capacity was never completely removed [40,41]. In our research, the immunoreactive proteins were not recognized in only one hydrolysate (SHB HVP), while, in the other two, they were found. Considering the molecular weight of immunoreactive peptides identified in hydrolysates, it may be accepted that they are allergenic subunits Gly m5 and Gly m6 that belong to the superfamily of cupine resistance to denaturation and hydrolysis. Around 40% of the European population allergic to soy had specific IgE to those allergens and 86% of the subjects with anaphylaxis were sensitized to those peptides [8]. Even a trace of these peptides in a product may cause an undesirable reaction in especially sensitive people.

Amigo-Benavent et al. [42], among others, investigated the effect of thermal processing on the allergenicity of soy proteins, who reported that protein antigenicity of soy products, which was estimated by immunoblotting against soy total antibodies, can be affected as a function of the intensity of thermal processing. Gomaa and Boye [43], using cookies with soy as an example, observed that, in general, allergen recovery decreased as baking time increased and cookie size was decreased. Van Boxtel et al. [44] observed that the combined effect of heating at 100 °C and pepsin hydrolysis for 10 min reduced the IgE-binding capacity of glycinin into a non-detectable capacity in immunoblot analysis.

Our research showed that almost half of the respondents are convinced that hydrolysis removes the immunoreactive properties of soy. Most of the interviewed medics claimed that patients allergic to soy may consume products with added soy preparations. While not questioning the lower allergenicity of hydrolysates compared to raw materials and considering their small amount in products, it must be remembered that allergic reactions can happen after contact with even a minimal amount of the allergenic content. Research on immunoreactive properties of soy preparations indicate the necessity to control individual additives and products. It is the only way to properly evaluate their antigenic properties and correctly label products, which, in turn, will increase the safety of allergic people.

## 6. Conclusions

It is necessary to educate consumers, especially allergic ones, in reading labels of food products.

Food technologists and medics think that hydrolysis removes immunoreactive properties, and that food products with the addition of soy preparations may be recommended to people with allergies. 

Analytical research shows that, to ensure the safety of people allergic to soy, it is necessary to control the allergenic properties of additives used. Isolates, concentrates, and even hydrolysates contain immunoreactive peptides that may be the source of hidden allergens that are dangerous to people of high sensitivity.

## Figures and Tables

**Table 1:** Characteristic of respondents taking part in the research (*n* = 251) and results of a chi-square independence test (*p*).

Factors	Categories of Features	E	M	F	Three Groups Together	*p*-Value
		(%)	(%)	(%)	(%)	
Sex	Women	37.6	34.0	24.0	32.3	NS
	Men	62.4	66.0	76.0	67.7	
Age	23–24 years	33.0	40.0	31.0	35.0	NS
	25–26 years	37.0	36.0	38.0	37.0	
	27–28 years	30.0	24.0	31.0	28.0	
Place of residence	Village	23.7	24.0	24.0	23.9	NS
	city < 200.000 citizens	42.3	52.0	44.0	45.0	
	city ≥ 200.000 citizens	33.7	24.0	32.0	31.1	
Food allergy		0	0	0	0	NS

NS-lack of significance at the α = 0.05.

**Table 2:** Respondents’ answers to selected questions from the questionnaire and results of the chi-square independence test (*p*).

Questions		% of Answers				Chi-Square Independence Test *p* Value
		E	M	F	Three Groups Together	
How often do you read the labels of the products you buy?	always	26.7	52.0	62.0	45.8	*p* < 0.001
	usually	55.4	44.0	38	46.2	
	never	17.9	4.0	0.0	8.0	
What is the sensitizing ingredient in soy?	protein	59.4	38.0	93.0	68.5	*p* < 0.001
	lipid	2.0	0.0	0.0	0.8	
	carbohydrates	5.0	22.0	2.0	7.2	
	I do not know	33.7	40.0	5.0	23.5	

**Table 3:** Respondents’ answers to questions depending on the possibility of reducing immunoreactive properties of soy and results of the chi-square independence test (*p*).

Processes That Can Reduce Soy’s Immunoreactivity		% of Answers				Chi-Square Independence Test *p* Value
		E	M	F	Three Groups Together	
Hydrolysis	yes	31.7	48	62	47	*p* < 0.001
	no	68.3	52	38	53	
Thermal treatment	yes	34.7	70	48	46	0.002
	no	65.3	30	52	54	
Fragmentation	yes	12.9	26	7	7	0.005
	no	87.1	74	93	93	
Genetic modification	yes	16.8	16	32	22.7	0.017
	no	83.2	84	68	77.3	
Pre-treatment (soaking)	yes	2	12	6	5.6	0.040
	no	98	88	94	94.4	
I do not know	yes	52.5	24	15	31.9	*p* < 0.001
	no	47.5	76	85	68.1	
